# Sustainable Cementitious Materials for Civil and Transportation Engineering

**DOI:** 10.3390/ma16186290

**Published:** 2023-09-19

**Authors:** Junjie Wang, Jianhe Xie, Yongliang Liu

**Affiliations:** 1 Department of Civil Engineering, Tsinghua University, Beijing 100084, China; 2 School of Civil and Transportation Engineering, Guangdong University of Technology, Guangzhou 510006, China; jhxie@gdut.edu.cn; 3 School of Mechanical and Aerospace Engineering, Jilin University, Changchun 130025, China; yliu2018@jlu.edu.cn

The current Special Issue entitled “Sustainable Cementitious Materials for Civil and Transportation Engineering” aims to discuss current research on the preparation, characterization, and application of sustainable cementitious materials for civil and transportation engineering, with a special focus on the development of low-carbon construction materials.

Concrete is the most widely used material for construction and building globally. The traditional cement and concrete industry is a main consumer of natural resources and contributes approximately 5~8% to total CO_2_ emissions. To address issues pertaining to sustainability and reduce the carbon footprint, it has become a new trend to research and develop novel sustainable cementitious materials, especially materials with low carbon emissions. In this context, on the one hand, construction and demolition waste (CDW) obtained from civil and transportation engineering, recycled concrete aggregates (RCAs) and recycled asphalt pavements (RAPs), for instance, could be treated and reused in the manufacturing of cementitious materials without compromising their properties, which would promote the sustainability of raw materials. 

The recycling of CDW [1,2] is a complex process, which usually includes the shredding, separation and processing of waste. First, the collected CDW is transported to crushing equipment for crushing. At present, common crushing equipment include jaw crushers, impact crushers, cone crushers, etc. During the crushing process, large pieces of the construction waste are broken into smaller blocks for subsequent separation and processing. Crushed construction wastes need to meet certain particle size distribution requirements in order to produce recycled aggregates, recycled sand and recycled powders that meet the requirements. After crushing, construction waste needs to be separated from different substances through a separation link in order to obtain purer recycled aggregates, recycled sand and recycled powder. Usually, separation is carried out via equipment such as vibrating screens and winnowing machines. A vibrating screen passes the crushed construction waste through a sieve with different pore sizes and divides it into aggregates and sand with different particle sizes. The winnowing machine uses the principle of aerodynamics to separate light substances such as plastic and paper from heavy substances such as bricks and concrete blocks. After separation, some impurities may remain in the construction waste, which need to be further processed. For example, magnetic separation equipment can be used to separate ferromagnetic substances such as steel bars, nails, etc.; selective flocculation equipment can separate suspended solids and soil in water.

After further processing, the construction waste could be converted into recycled aggregates, recycled sand and recycled powder. In the recycling process, it is necessary to control the particle size distribution, particle shape, impurity content and other indicators of recycled aggregates, recycled sand and recycled powder to ensure that their quality and performance meet the requirements of concrete manufacturing.

Portland cement is a main source of carbon emissions; on the other hand, Portland cement could be partially or completely replaced with recycled cement [3,4], industrial solid waste, clay minerals, limestone, etc., to produce low-carbon binders such as low-clinker cement (i.e., limestone calcined clay cement (LC^3^), calcium aluminate cement, and calcium sulfoaluminate cement) and alkali (acid)-activated binders (i.e., geopolymer) [5,6]. For example, 50 wt.% clinker can be replaced with limestone and calcined clay to prepare LC^3^ of comparable strength, but it can reduce CO_2_ emissions 20–40%, costs by 15–25%, and thermal energy requirements by 20%. Moreover, geopolymer is an alkali-activated binder with excellent properties and is a promising alternative to Portland cement. During the preparation of geopolymer concrete, solid waste and an alkali activator that is derived from the waste are frequently used, and therefore the carbon footprint can be further reduced. Successful applications of these sustainable cementitious materials in civil and transportation engineering are also significant.

The research interests of this Special Issue include, but are not limited to, the following: low-carbon cementitious binders; carbonation-enhanced concrete; low-carbon cement and concrete technology based on non-Portland cement systems, such as alkali-activated materials or geopolymeric materials; recycled aggregate concrete; green admixtures for cement and concrete; the durability of low-carbon concrete; case studies on applications of sustainable cementitious materials in specific regions for civil and transportation engineering.
